# The association of food consumption and nutrient intake with endometriosis risk in Iranian women: A case-control study

**DOI:** 10.18502/ijrm.v17i9.5102

**Published:** 2019-09-22

**Authors:** Youseflu Samaneh, Sadatmahalleh ShahidehJahanian, Mottaghi Azadeh, Kazemnejad Anoshirvan

**Affiliations:** ^1^Department of Reproductive Health and Midwifery, Faculty of Medical Sciences, Tarbiat Modares University Tehran Iran.; ^2^Research Center for Prevention of Cardiovascular Diseases, Institute of Endocrinology and Metabolism, Iran. University of Medical Sciences Tehran Iran.; ^3^Department of Biostatistics, Faculty of Medical Sciences, TarbiatModares University Tehran Iran.

**Keywords:** Endometriosis, Macronutrient, Diet, Case-control study

## Abstract

**Background:**

Endometriosis, defined as the attendance of endometrial-like lesions in extra uterine locations, causes pain, infertility, and reduced quality of life.

**Objective:**

To evaluate the relationship between food consumption and nutrient intake with risk of endometriosis.

**Materials and Methods:**

Of the 156 women approached for the study, 78 women had endometriosis and 78 healthy women were included in the control group. Dietary data were collected using a validated 147-item semi-quantitative Food Frequency Questionnaire (FFQ) with the standard serving size. A logistic regression model was used to determine the association of macronutrients and energy intake with the risk of endometriosis.

**Results:**

In women with higher intake of protein, especially animal protein, monounsaturated fatty acids, soluble and insoluble fiber, oleic acid, eicosapentaenoic acid, and docosahexaenoic acid endometriosis is less common (p< 0.05). High consumption of vegetables, fruits, red meat, yellow vegetables, potatoes, legumes, dairy products, liquid oil, and low intake of fried potatoes was associated with a lower risk of endometriosis (p< 0.05).

**Conclusion:**

Regarding the association of dietary intake on endometriosis risk, counseling about improving the dietary structure can contribute toward the prevention and control of endometriosis.

## 1 Introduction

***This article extracted from M.Sc. Thesis. (Samaneh Youseflu)***

Endometriosis is defined as the attendance of endometrial glands and stroma in extrauterine locations; it causes pain, infertility, and reduced quality of life (1). Certain diagnosis of endometriosis is performed by using laparoscopic surgery (2). The accurate prevalence of endometriosis is secret because there no noninvasive tool for diagnosing this disease. Endometriosis affects approximately 2.5–3.3% of reproductive-age women (3), however, in women with pelvic pain and/or infertility, its prevalence ranges from 30 to 50% (4). A common theory for the explanation of endometriosis is as follows: “the reflux of endometrial tissue flows through the fallopian tubes into the peritoneal cavity” (5). The etiology of endometriosis is complex and multifaceted that involves hormonal, genetic and immunologic mechanisms, contraction of the smooth muscle and inflammatory factors, as well as anatomic and environmental agents such as diet and exercise (6). Agents that impress the volume of retrograde menstruation or affect a woman’s aptitude for the implantation of the endometriotic lesion are of etiological fondness. Scientific research has proposed that estrogen without progesterone may enhance the endometriosis risk (7).

Some dietary components could modulate endogenous hormone metabolism as well as demonstrate or imitate estrogen; for example, consumption of fat, phytoestrogen, coffee, fiber, and alcohol has been related to endogenous estrogen levels. Fatty acids of diet can modify inflammatory markers (8). Dairy products contain estrogen, progesterone, anti-tumorigenic and anti-inflammatory ingredients, calcium, vitamin D, butyric acid, and polyunsaturated fatty acids (PUFAs) (9). Consumption of fruits and vegetables rich in different antioxidants, phytochemicals, and anti-carcinogenic substances can modify the function of the immune system and ruin the free radicals (10, 11).

The objective of the present study was to compare the dietary intake of fruits and vegetables, cereals, meats, oils, dairy products, and other macronutrients in women with and without pelvic endometriosis.

## 2 Materials and Methods

This case-control study was conducted between May 2016 and February 2017 on women with endometriosis at the Infertility Clinic of Arash Hospital in Tehran, Iran. The total number of patients who indications for laparoscopy during the study was allocated. Those women who had abnormalities other than endometriosis at laparoscopy (such as adhesions, fibroids, leiomyomas, and/or uterine abnormalities) were excluded from the study. A total of 156 women was included in the study. They were divided according to the laparoscopy findings into two groups (n= 78/each): a case group consisting of women with visual lesions of endometriosis and a control group including women no visual lesions of endometriosis. Women in the two groups were comparable in demographic and personal characteristics.

The most common indications for laparoscopy were as follows: symptoms of endometriosis, such as dyspareunia, dysmenorrhea and pelvic pain, unexplained factor in infertility, uterine abnormality and tubo-peritoneal disorder. After laparoscopy, we divided the 1282 participants into three groups, shown in the diagram. The first group of 341 patients had visual lesions of endometriosis (52 had stage I, 85 had stage II, 111 had stage III, and 84 had stage IV); the second group of 609 patients had adhesions, fibroids, leiomyomas, and/or uterine abnormalities; the third group of 332 subjects had no visual lesions of endometriosis, i.e., a normal pelvis without any complications. In analysis, the second group was excluded in order to evaluate the risk factors in women with endometriosis (pure endometriosis) compared with the group with a normal pelvis and no other complications.

The inclusion criteria included age between 15 and 45 years, the absence of the history of chronic diseases, Iranian race, no previous pregnancy, not using medication affecting the adsorption of food, appetite, and basal metabolism of the body, no smoking and lack of mental retardation.

In the beginning, a socio-demographic questionnaire including questions about socio-economic status was completed. Marital status, age, smoking, education, habitat, physical activity, ethnicity, anthropometric measures, obstetrics and gynecology characteristics, and medical histories were completed by women. Then the dietary information was completed by the researcher.

### 2.1 Assessment of diet

In the face-to-face interviews by trained staff, a common dietary intake during the past year was assessed by 147-item semi-quantitative Food frequency questionnaire (FFQ) (12). This questionnaire includes a number of dietary items from usual Iranian foods with standard serving measures. The Persian version of FFQ has previously been evaluated for both reliability and validity (12).

``The participants were asked to report their usual food intake during the previous year on a daily, weekly, monthly, and yearly basis; all these were converted to daily intakes." By using domestic measurement (13), portion sizes of the consumed food were transformed to grams. All consumed dietary items were analyzed for their energy and nutrient components using a nutrient database (Nutritionist ш, Mosby Nutritract software, and ver.7.0, N-Squared Computing, Salem, OR, USA), which was modified according to the Iranian Food Composition Table (FCT) (14) because the Iranian FCT is incomplete. We excluded individuals who reported unusual total energy intake levels (more than 4,300 kcal or less than 670 kcal).

### 2.2 Ethical consideration

This study was approved by the Ethics Committee of TarbiatModares University of Medical Sciences (IR.TMU.REC.1395.358). All women participated voluntarily and provided a signed informed consent.

### 2.3 Statistical analysis

Statistical analysis of the obtained data was performed by using the Statistical Package for the Social Sciences, version 21, SPSS Inc., Chicago, Illinois, USA (SPSS). The K–S test was selected to check the normality of socio-demographic characteristics. Comparisons between the two groups were done using *t*-Test, Mann–Whitney, and Chi-square tests, and p< 0.05 was considered statistically significant. For the different variables, odds ratio (OR; adjusted for age, total energy intake, BMI, and income) and 95% confidence intervals (CIs) were calculated using logistic regression models to assess the strength of the associations between the intake of food groups or macro-nutrients and the risk of endometriosis. Each dietary variable and macro-nutrient was divided into four groups using quartile of intake based on the distribution of control subjects. To calculate the linear trend in the odds of dietary variable quartile, median factor score of each quartile was entered into the logistic regression analysis. Quartile 1 served as the reference category for all regression analyses.

## 3 Results

In this study, we collected dietary data from 78 women with endometriosis and 78 controls. The mean age of women with endometriosis and control group were 31.01±6.56 and 29.35±7.00 yr, respectively; 32 (41%) women with endometriosis and 41 (52.5%) controls had pervious parities. Among women with endometriosis, 50 (64.1%) had a BMI between 18.5 and 24.9, 24 (30.8%) between 25 and 29.9, and 4 (5.1%) had more than 30. The mean menarche age and the age at the first pregnancy of case group was 13.49±2.38 and 23.26 ± 5.45 yr, respectively. There were no statistically significant difference in the women’s age, BMI, parity, education, occupation, income, and age at menarche between the two groups (p > 0.05; Table I).

**Table I:** Demographic and anthropometric characteristics of women with and without endometriosis

Characteristic	Case group (N = 78)	Control group (N = 78)	P-value
Age (yr)^*^	31.01 ± 6.56	29.35 ± 7.00	0.13
BMI^**^			
< 25	50 (64.1)	50 (64.10)	
25–29.9 (over weight)	24 (30.8)	24 (30.77)	0.19
≥ 30 (obese)	4 (5.1)	4 (5.13)	
Education^**^			
Lower than university	42 (53.8)	38 (48.72)	0.58
University	36 (46.2)	40 (51.28)	
Age at menarche^***^	13.49 ± 2.38	13.35 ± 1.64	0.70
Parity^**^			
Parous	32 (41)	41 (52.56)	0.06
Nulliparous	46 (59)	35 (44.87)	
Age at first pregnancy^***^	23.26 ± 5.45	22.25 ± 3.97	0.69
Occupation^2^			
Housewife	61 (78.2)	68 (87.18)	0.09
Employed	17 (21.8)	10 (12.82)	

* Values are given as mean ± SD using Student’s *t*-test; **values are given as number (%) using Chi-squared test; ***values are given as mean ± SD using Mann–Whitney’s test

BMI: Body mass index

Table II summarizes the ORs for endometriosis by daily nutrient intakes such as energy, type of protein, carbohydrate, and selected fatty acids according to the quartile of intake. The consumption of total protein showed no significant relationship between the two groups, but in the women with higher protein intake (fourth quartile OR: 0.36; %95CI: 0.14–0.91; P-trend= 0.06), especially animal protein (OR: 0.37; %95CI: 0.29–0.95; P-trend= 0.02), endometriosis is less common. There was no significant difference between the two groups for received total fat, but the consumption of eicosapentaenoic acid (EPA) (OR: 0.71, %95CI: 00.0–0.92, P-trend= 0.04) and docosahexaenoic acid (DHA) (OR: 0.70; %95CI: 0.00–0.95; P-trend= 0.04) was statistically significant between the two groups. We observed inverse associations between the consumption of monounsaturated fatty acids (MUFAs) and endometriosis risk in the third quartile (OR: 0.28; %95CI: 0.12–0.79; P-trend= 0.60). In the case group, oleic acid intake in the third quartile was less than the control group (third quartile OR: 0.30; %95CI: 0.11–0.71; P-trend= 0.64).

**Table II:** . Adjusted odds ratios (OR) of endometriosis and corresponding 95% confidence intervals (CI) according to nutrient intake

Quartile						
Nutrient item	1	2 OR (95% CI)	3 OR (95% CI)	4 OR (95% CI)	Total* OR (95% CI)	p-trend
Energy (kcal)						
OR (95% CI) Case/control	1.00 22/16	0.80 (0.33–1.97) 21/19	0.92 (0.73–1.60) 18/21	0.90 (0.58–1.35) 17/21	0.99 (0.99–1.00)	0.94
Protein (g)						
OR (95% CI) Case/control	1.00 24/15	0.66 (0.27–1.62) 20/19	0.61 (0.24–1.66) 21/16	0.36 (0.14–0.91) 14/24	0.48 (0.45–1.00)	0.06
Vegetable protein (g)						
OR (95% CI) Case/control	1.00 18/20	1.00 (0.96–1.04) 21/19	1.02 (0.90–1.07) 19/19	1.02 (0.94–1.04) 20/19	0.98 (0.95–1.03)	0.56
Animal protein (g)						
OR (95% CI) Case/control	1.00 22/17	0.90 (0.3–1.79) 19/20	0.67 (0.55–3.43) 25/14	0.36 (0.14–0.91) 12/26	0.37 (0.29–0.95)	0.02
Saturated fatty acids (g)						
OR (95% CI) Case/control	1.00 21/18	0.71 (0.65–1.17) 24/15	1.00 (0.92–1.14) 18/21	1.16 (0.95–1.06) 15/23	0.98 (0.95–1.02)	0.35
Oleic acid (g)						
OR (95% CI) Case/control	1.00 26/13	0.79 (0.35–1.26) 17/22	0.30 (0.11–0.71) 14/25	0.62 (0.24–1.55) 21/17	0.98 (0.95–1.04)	0.64
Total fat (g)						
OR (95% CI) Case/control	1.00 23/16	0.79 (0.30–1.79) 19/20	0.39 (0.16–1.02) 25/14	0.77 (0.35–2.12) 12/16	0.99 (0.99–1.01)	0.60
Mono unsaturated fatty acids (g)						
OR (95% CI) Case/control	1.00 25/14	0.83 (0.19–1.19) 18/21	0.28 (0.12–0.79) 14/25	0.69 (0.27–1.73) 21/17	0.98 (0.94–1.06)	0.60
Linoleic acid (g)						
OR (95% CI) Case/control	1.00 14/7	0.83 (0.17–1.46) 23/23	0.36 (0.13–1.01) 29/40	0.86 (0.23–3.15) 12/7	0.98 (0.93–1.04)	0.50
Linolenic acid (g)						
OR (95% CI) Case/control	1.00 22/17	0.77 (0.31–1.89) 19/19	0.67 (0.28–1.61) 19/22	0.73 (0.29–1.81) 18/19	0.89 (0.55–1.42)	0.62
Poly unsaturated fatty acids (g)						
OR (95% CI) Case/control	1.00 25/14	0.56 (0.17–1.08) 16/23	0.54 (0.22–1.32) 18/21	0.69 (0.28–1.72) 20/18	0.99 (0.96–1.03)	0.46
Cholesterol (mg)						
OR (95% CI) Case/control	1.00 23/16	0.54 (0.22–1.32) 17/22	0.60 (0.24–1.46) 18/21	0.77 (0.31–1.90) 20/18	1.00 (0.99–1.00)	0.72
EPA (g)^1^						
OR (95% CI) Case/control	1.00 24/17	1.04 (0.42–2.56) 22/15	0.59 (0.25–1.39) 20/24	0.40 (0.16–1.04) 20/24	0.71 (0.00–0.92)	0.04
DHA (g) ^2^						
OR (95% CI) Case/control	1.00 23/21	1.60 (0.68–3.75) 28/16	0.62 (0.25–1.57) 13/19	0.61 (0.25–1.49) 14/21	0.70 (0.00–0.95)	0.04
Trans fat (g)						
OR (95% CI) Case/control	1.00 36/34	1.13 (0.49–2.16) 18/15	0.66 (0.23–1.93) 7/10	0.89 (0.39–2.01) 17/18	0.98 (0.78–1.37)	0.55
Carbohydrate (g)						
OR (95% CI) Case/control	1.00 21/18	1.11 (0.45–2.71) 22/17	0.48 (0.19–1.19) 14/25	1.06 (0.43–2.60) 21/17	1.00 (0.99–1.00)	0.35
Total fiber (g)						
OR (95% CI) Case/control	1.00 22/17	0.76 (0.41–2.45) 22/17	0.38 (0.19–1.19) 14/25	0.88 (0.31–1.89) 21/17	0.45 (0.36–1.03)	0.07
s. fiber (g) ^3^						
OR Case/control	1.00 24/14	0.52 (0.21–1.30) 19/21	0.29 (0.18–1.09) 19/21	0.44 (0.18–1.12) 16/21	0.33 (0.11–0.99)	0.04
i.fiber (g) ^4^						
OR (95% CI) Case/control	1.00 26/14	0.42 (0.17–1.03) 17/22	0.48 (0.19–1.20) 18/20	0.44 (0.17–1.08) 17/21	0.76 (0.59–0.99)	0.04
c.fiber (g) ^5^						
OR Case/control	1.00 23/16	0.66 (0.27–1.62) 19/20	0.73 (0.30–1.79) 20/19	0.51 (0.20–1.25) 20/19	0.97 (0.91–1.02) 16/22	0.27
Total sugar (g)						
OR Case/control	1.00 22/17	0.89 (0.39–2.01) 19/20	0.60 (0.24–1.46) 17/22	0.86 (0.35–2.11) 20/18	0.99 (0.98–1.00)	0.38

* Odds ratio adjusted for age, energy intake, BMI, income. Quartile 1 as reference categories

1- Eicosa pentaenoic acid; 2-Docosahexaenoicacid;3-soluble fiber; 4- insoluble fiber; 5- crude fiber

In the women with endometriosis, the intake of soluble (OR: 0.33; %95CI: 0.11–0.99; P-trend = 0.04) and insoluble fibers (OR: 0.76; %95CI: 0.59–0.99; P-trend = 0.04) was significantly less than the control group, but this relationship was not seen in total fiber consumption (p > 0.05).

Table III demonstrates the OR of endometriosis and the corresponding 95% CIs according to the quartiles of daily food intake. A high consumption of vegetables in fourth quartile was associated with a lower risk of endometriosis (OR: 0.38; %95CI: 0.15–0.96; P-trend = 0.25), but this difference was more related to the consumption of yellow vegetables (OR: 0.56; %95CI: 0.47–0.87; P-trend = 0.03). An increased consumption of potatoes as one of the starchy vegetables was associated with a reduced risk of endometriosis (third quartile OR: 0.33; %95CI: 0.13–0.85, fourth quartile OR: 0.40; %95CI: 0.16–0.99; P-trend = 0.11), but a significant increase in the risk of endometriosis was found for fried potatoes (Third Quartile OR: 4.13; %95CI: 1.6–10.66; P-trend= 0.19).

**Table III:** . Adjusted odds ratios (OR) of endometriosis and corresponding 95% confidence intervals (CI) according to the daily intake of food

Quartile						
Food intake	1	2 OR (95% CI)	3 OR (95% CI)	4 OR (95% CI)	Total OR (95% CI)	P-trend
Vegetables						
OR (95% CI) Case/control	1.00 24/14	0.61 (0.18–1.12) 17/22	0.67 (0S.3–1.88) 22/17	0.38 (0.15–0.96) 15/23	0.70 (0.59–1.03)	0.25
Green Vegetables						
OR (95% CI) Case/control	1.00 25/14	0.43 (0.34–1.07) 17/22	0.48 (0.19–1.19) 18/21	0.50 (0.20–1.26) 18/20	0.62 (0.49–1.01)	0.10
Yellow Vegetables						
OR (95% CI) Case/control	1.00 23/16	0.85 (0.35–2.07) 22/18	0.56 (0.23–1.39) 17/21	0.50 (0.20–1.25) 16/22	0.56 (0.47–0.87)	0.03
Fruit						
OR (95% CI) Case/control	1.00 25/14	0.84 (0.18–1.13) 35/42	0.70 (0.32–2.01) 23/16	0.29 (0.11–0.74) 13/25	0.58 (0.44–1.01)	0.10
Cereal						
OR (95% CI) Case/control	1.00 16/22	1.78 (0.72–4.39) 22/17	1.45 (0.59–3.56) 20/19	1.37 (0.56–3.4) 20/19	1.00 (0.99–1.00)	0.65
Legumes						
OR (95% CI) Case/control	1.00 28/13	0.43 (0.22–1.26) 25/22	0.23 (0.09–0.60) 12/24	0.41 (0.12–0.83) 12/18	0.49 (0.36–0.72)	0.02
Red meat						
OR (95% CI) Case/control	1.00 25/13	0.81 (0.33–1.99) 19/23	0.65 (0.35–1.05) 20/16	0.36 (0.14–0.91) 14/25	0.70 (0.59–1.03)	0.09
Poultry						
OR (95% CI) Case/control	1.00 27/26	0.69 (0.31–1.56) 18/25	0.84 (0.34–2.07) 14/16	1.83 (0.80–4.66) 19/10	0.98 (0.98–1.01)	0.59
Fish						
OR (95% CI) Case/control	1.00 20/16	1.17 (0.46–2.97) 22/15	0.67 (0.27–1.61) 20/24	0.46 (0.17–1.2) 12/21	0.71 (0.44–1.00)	0.08
Egg						
OR (95% CI) Case/control	1.00 19/20	1.05 (0.44–2.54) 20/20	1.23 (0.50–2.99) 21/18	0.99 (0.40–2.45) 18/19	0.98 (0.96–1.01)	0.49
Total dairy product						
OR (95% CI) Case/control	1.00 23/16	0.81 (0.33–1.99) 21/18	0.96 (0.38–2.37) 22/16	0.32 (0.13–0.82) 12/26	0.46 (0.39–0.93)	0.02
Low–fat dairy foods						
OR (95% CI) Case/control	1.00 24/15	0.89 (0.19–1.19) 17/22	0.90 (0.39–1.01) 17/22	1.01 (1.00–1.02) 20/18	0.98 (0.97–1.01)	0.42
High–fat dairy foods						
OR (95% CI) Case/control	1.00 21/18	1.52 (0.62–3.8) 25/14	0.73 (0.30–1.79) 18/21	0.50 (0.20–1.24) 14/24	0.71 (0.58–1.01)	0.11
Potato						
OR (95% CI) Case/control	1.00 26/15	0.77 (0.32–1.56) 24/18	0.33 (0.13–0.85) 12/21	0.40 (0.16–0.99) 16/23	0.79 (0.76–1.00)	0.11
Fried potatoes						
OR (95% CI) Case/control	1.00 17/26	1.17 (0.52–2.66) 23/30	4.13 (1.6–10.66) 27/10	1.53 (0.54–4.31) 11/11	1.19 (0.88–1.00)	0.19
Olive oil						
OR (95% CI) Case/control	1.00 39/37	0.95 (0.13–7.09) 2/2	0.90 (0.41–1.97) 18/19	0.95 (0.43–2.07) 19/19	0.98 (0.92–1.16)	0.10
Hydrogenated vegetable oil						
OR (95% CI) Case/control	1.00 35/38	0.22 (0.02–1.95) 1/5	1.18 (0.57–2.42) 26/24	1.74 (0.70–4.33) 16/10	0.99 (0.98–1.01)	0.52
Liquid oil						
OR (95% CI) Case/control	1.00 47/34	1.00 0/0	0.42 (0.19–0.90) 15/26	0.68 (0.30–1.53) 16/17	0.98 (0.97–1.07)	0.21
Nuts						
OR (95% CI) Case/control	1.00 21/18	1.06 (0.43–2.59) 21/17	0.81 (0.33–1.98) 19/20	0.62 (0.25–1.53) 16/22	0.98 (0.96–1.01)	0.23

* Odds ratio adjusted for age, energy intake, BMI, income;Quartile 1 as reference categories

The consumption of fruit in the upper quartile was associated with a significantly decreased endometriosis risk (fourth quartile OR: 0.29; %95CI: 0.11–0.74; P-trend= 0.10). A higher consumption of legumes was inversely associated with endometriosis risk (OR: 0.49; %95CI: 0.36–0.72, P-trend= 0.02). The consumption of red meat in the fourth quartile was associated with a lower risk for endometriosis (fourth quartile OR: 0.36; %95CI: 0.14–0.91; P-trend=0.09). The individuals in the highest quartile of total dairy products, consumption had a significantly lower risk of endometriosis than those in the lowest quartile (OR: 0.46; %95CI: 0.39–0.93; P-trend= 0.02). However, this relationship was not seen in the type of dairy products (low-fat or high-fat). The consumption of liquid oil in the third quartile was associated with reduced risk of endometriosis (third quartile OR: 0.42; %95CI: 0.19–0.90; P-trend= 0.21).

## 4 Discussion

Our findings suggest that higher consumption of vegetables, especially yellow vegetables, dairy products, fruits, legumes, red meat, potato, and liquid oil was associated with reduced endometriosis risk in women, whereas positive associations were found for the consumption of fried potatoes and endometriosis risk. Regarding nutrient intake, protein, especially animal protein, EPA, MUFA, and oleic acid, as well as soluble and insoluble fibers were related to a decreased risk of endometriosis.

Only in the upper quartiles, protein intake was associated with a reduced risk of endometriosis. By dividing the proteins into two groups of animal and plant origin, this association was seen only in the consumption of animal-based proteins. Eggs, dairy products, fish, and meat are the main sources of meat-based proteins. Dietary sources of vegetable protein include cereals, legumes, and nuts. The body requires the protein for the production of hormones, enzymes, antibodies, and hemoglobin. Net protein synthesis is more with a high animal-protein diet than with a high vegetable-protein diet. Plant-based foods for proteins are deficient in many types of essential amino acids, including lysine, threonine. Methionine, tryptophan, glycine, and cysteine (15). Eggs are rich sources of bioactive proteins that are effective in the down-regulation of cytokine-induced inflammatory proteins and reduction of oxidative stress (16). Casein and whey protein in milk products have anti-inflammatory properties as well as immunomodulation and anticarcinogenic activity (17). High-protein diet helps improve lipid profile and weight loss (18). Therefore, a high protein diet is effective on the immune and hormonal systems. Inflammatory response and weight loss can be related to endometriosis risk.

In the current study, the total fat intake was not associated with endometriosis risk. Following the classification of fats, this statistical relationship was found only in the consumption of MUFAs (in the third quartile), especially oleic acid for the third quartiles, DHA, and EPA.

The consumption of MUFA, oleic acid, DHA, and EPA was associated with decreased CRP levels and suppression of immune cell functions (19,20). In this regard, the results of Trabert and colleagues' study (21) showed that more consumption of total fats, MUFAs, and dairy products was associated with a decrease in the risk of endometriosis. On the contrary, in the study by Britton and colleagues (22), a high intake of vegetable fat and MUFAs was associated with an increased risk of endometriosis. While Missmer and co-workers (23) did not observe a significant association between the total fat consumption and endometriosis risk, a high intake of Omega-3 was linked to a reduced risk of endometriosis, and a high intake of trans-unsaturated fat was associated with an increased risk of endometriosis.

In this study, the total fiber intake was not significantly different between the two groups, but both soluble and insoluble fibers’ intake in the case group was less than in the control group. Experimental studies have shown that a high-fiber diet has a down-regulation role on inflammation and reduces CRP level (24). A diet rich in fiber could elevate SHBG levels (25) and thus decrease the level of bioavailable estrogens. On the contrary, in the study of Savaris, high dietary fiber-intake was associated with an increased risk of endometriosis (26). In other studies, there was no significant relationship between dietary fiber intake and the risk of endometriosis.

In this study, an increased consumption of vegetables and fruits in the fourth quartile was related to a reduced risk of endometriosis, but the difference was related to the consumption of yellow vegetables. A high consumption of vegetables and fruits was associated with an improved serum antioxidant status and lower concentrations of CRP (27). Similarly, in Parazzini’s study, an increased intake of vegetables was associated with a reduction in the risk of endometriosis (28). On the contrary, in the study of Trabert, a high consumption of fruits was significantly associated with an increased risk for endometriosis (21).

The data showed that the consumption of potato was associated with a reduced risk of endometriosis; however, eating fried potatoes increased the risk of endometriosis. Potatoes contain plant phenols and antioxidant compounds, which improve the level of lipids and lipoproteins in the blood; thus, they have anti-inflammatory properties and reduce the concentration of CRP and IL6 (29).

High carbohydrate foods fried at high temperatures lead to the release of carcinogens like acrylamide. Dietary acrylamide in fried potatoes causes decreased serum level of total high-density lipoprotein and total testosterone and increases the production of reactive oxygen species, CRP, interleukin-6, and γ-glutamyltransferase (30). In our study, an increased consumption of red meat in the fourth quartile was associated with a reduced risk of endometriosis. Red meat is a rich source of high biological components such as protein, iron, vitamin B12, folic acid, zinc, selenium, and phosphorus.

Taurine and PUFAs in animal meats have antioxidant and anti-inflammatory effects (31). In Trabert and colleagues' study, there was no significant relationship between the consumption of red meat and endometriosis risk. However, the meat intake was slightly higher in the control group (21). In the study of Parazzini and Yamamoto, an increased consumption of red meat was significantly associated with an increased risk of endometriosis (28, 32). Average meat consumption in Iranian population is much lower than in the Italian and American population. Another difference is that there has been dioxin contamination in foodstuff and animal food in Italy during the above study period, which could be effective in the study results (33). In addition, the way of cooking meat can be highly effective in its components (34), and we know that different nations and cultures have different cooking habits/styles. Our findings in this study showed a decreased risk of endometriosis with increased intake of legumes. Legume consumption was inversely associated with the serum concentrations of CRP, TNFα, IL-6, and other adhesion molecules, as well as the level of adiponectin (35). Regarding the inflammatory nature of endometriosis, legumes can play an important role in the prevention and control of this disease. In this study, women with endometriosis compared with healthy women consumed less dairy products; however, there was no significant difference between the two groups regarding the type of dairy products (low-fat or high-fat). High dairy consumption inhibits oxidative and inflammatory biomarkers (36), so it can reduce the risk of endometriosis. In a prospective cohort study by Harris and co-worker (37), an increased low-fat dairy consumption (more than three units) was associated with 18% reduction in the risk of endometriosis. Similarly, in Trabert and colleagues' study (21), increased consumption of dairy products was associated with decreased risk of endometriosis. In a case-control study by Parazzini and co-worker (28), no association emerged between the risk of endometriosis and increasing the servings of milk and cheese.

### 4.1 Limitations of the studys

The main limitation of the present study was the problem of persuading the subjects to answer a lot of questions. Also because of the case-control study design, the possibility of selection bias and recall bias including under- or over-reporting of the specific dietary item might have affected our results.

## 5 Conclusion

Regarding the influence of dietary components on endometriosis risk, counseling about improving the dietary structure can contribute to the prevention and control of endometriosis.

## Conflict of Interest

There are no conflicts of interest to declare.
