# Corrigendum to “The association of food consumption and nutrient intake with endometriosis risk in Iranian women: A case-control study” [Int J Reprod BioMed 2019; 17: 661-670]

**DOI:** 10.18502/ijrm.v20i11.12366

**Published:** 2022-12-10

**Authors:** Samaneh Youseflu, Shahideh Jahanian Sadatmahalleh, Azadeh Mottaghi, Anoshirvan Kazemnejad

The authors have been informed of an error that occurred on page 661 in which the word “Iran” has been missed in the affiliation of the third author (Azadeh Mottaghi), which should be corrected as: “Research Center for Prevention of Cardiovascular Diseases, Institute of Endocrinology and Metabolism, Iran University of Medical Sciences, Tehran, Iran”. On behalf of the author, the publisher wishes to apologize for this error. The online version of article has been updated on 15 November 2022 and can be found at https://doi.org/10.18502/ijrm.v17i9.5102. How

